# Fabrication, Characterization, and Evaluation of Bionanocomposites Based on Natural Polymers and Antibiotics for Wound Healing Applications

**DOI:** 10.3390/molecules21060761

**Published:** 2016-06-10

**Authors:** Marius Rădulescu, Alina Maria Holban, Laurențiu Mogoantă, Tudor-Adrian Bălşeanu, George Dan Mogoșanu, Diana Savu, Roxana Cristina Popescu, Oana Fufă, Alexandru Mihai Grumezescu, Eugenia Bezirtzoglou, Veronica Lazar, Mariana Carmen Chifiriuc

**Affiliations:** 1 Department of Inorganic Chemistry, Physical Chemistry and Electrochemistry, Faculty of Applied Chemistry and Materials Science, University Politehnica of Bucharest, 1–7 Polizu Street, 011061 Bucharest, Romania; radulescu_marius@yahoo.com; 2 Department of Science and Engineering of Oxide Materials and Nanomaterials, Faculty of Applied Chemistry and Materials Science, University Politehnica of Bucharest, 1–7 Polizu Street, 011061 Bucharest, Romania; alina_m_h@yahoo.com (A.M.H.); roxpopescu@yahoo.co.uk (R.C.P.); oana.fufa@gmail.com (O.F.); grumezescu@yahoo.com (A.M.G.); 3 Microbiology Immunology Department, Faculty of Biology, University of Bucharest, 1–3 Portocalelor Lane, Sector 5, 77206 Bucharest, Romania; veronica.Lazar2009@gmail.com (V.L.); carmen_balotescu@yahoo.com (M.C.C.); 4 Research Institute of the University of Bucharest, Life, Environmental and Earth Sciences, Spl. Independentei 91–95, 0500088 Bucharest, Romania; 5 Research Center for Microscopic Morphology and Immunology, University of Medicine and Pharmacy of Craiova, PetruRares Street, No. 2, 200349 Craiova, Romania; editor@rjme.ro; 6 Research Center for Clinical and Experimental Medicine, University of Medicine and Pharmacy of Craiova 2 PetruRareş Street, 200349 Craiova, Romania; adibalseanu@yahoo.com; 7 Department of Pharmacognosy & Phytotherapy, Faculty of Pharmacy, University of Medicine and Pharmacy of Craiova, PetruRares Street, No. 2, 200349 Craiova, Romania; mogosanu2006@yahoo.com; 8 Department of Life and Environmental Physics, “HoriaHulubei” National Institute of Physics and Nuclear Engineering, Magurele, 077125 Bucharest, Romania; dsavu@nipne.ro; 9 Lasers Department, National Institute for Laser, Plasma and Radiation Physics, Magurele, 077125 Bucharest, Romania; 10 Laboratory of Microbiology, Biotechnology and Hygiene, Department of Food Science and Technology, Faculty of Agricultural Development, Democritus University of Thrace, 68200 Orestiada, Greece

**Keywords:** chitin, sodium alginate, drug delivery, cefepime, cefuroxime, L929 cell line, rats, anti-biofilm

## Abstract

The aim of our research activity was to obtain a biocompatible nanostructured composite based on naturally derived biopolymers (chitin and sodium alginate) loaded with commercial antibiotics (either Cefuroxime or Cefepime) with dual functions, namely promoting wound healing and assuring the local delivery of the loaded antibiotic. Compositional, structural, and morphological evaluations were performed by using the thermogravimetric analysis (TGA), scanning electron microscopy (SEM), and fourier transform infrared spectroscopy (FTIR) analytical techniques. In order to quantitatively and qualitatively evaluate the biocompatibility of the obtained composites, we performed the tetrazolium-salt (MTT) and agar diffusion *in vitro* assays on the L929 cell line. The evaluation of antimicrobial potential was evaluated by the viable cell count assay on strains belonging to two clinically relevant bacterial species *(i.e.*, *Escherichia coli* and *Staphylococcus aureus)*.

## 1. Introduction

The genuine features (such as high surface/volume ratio, unique physicochemical and biological properties) and unprecedented functionalization versatility of polymer-based nanostructures strongly suggest their suitability as ideal candidates for drug delivery [1,2,3,4,5].The scientific community has turned its attention especially towards the renewable naturally derived macromolecular compounds.

Chitin is a linear macromolecular structural component found within the cell walls of various microorganisms (yeasts and molds) [6,7,8,9]), exoskeleton of insects [10,11,12] and crustaceans [13,14,15,16]), and internal shells of cephalopods [17,18,19,20]. It is one of the most abundant naturally derived polysaccharides formed by the β (1 → 4) linkage of *N*-acetyl-2-amino-2-deoxy-d-glucose and 2-amino-2-deoxy-d-glucose monomeric units, which specifically provide chitin with a rigid and semi-crystalline fiber-like structure that is insoluble in water and other common solvents [21,22,23]. Since the fundamental extracellular matrix of multicellular organisms contains significant amounts of *N*-acetyl glucosamine within the glycosaminoglycans [24,25,26], chitin and chitin-derivatives are non-harmful for biological systems (in terms of biocompatibility, biodegradability, and non-toxicity) [27,28]. Given the physicochemical versatility and biological behavior of chitin, novel nanotechnology-derived materials and systems based on chitin have been successfully developed for potential biomedical applications, including biosensing [29,30] and bioimaging [31,32,33], tissue engineering [34,35,36], and drug delivery [37,38,39].

Regarding nanotechnology-related drug delivery applications, chitin and its derivatives have attracted remarkable attention from the scientific community. Thus, considering the challenges of current therapies (in terms of drug resistance and systemic or topic side effects) and the processing versatility of chitin-derived compounds, various research studies have reported successful data regarding chitin-based nanostructures for controlled and/or targeted delivery of antibiotics [40,41,42], antivirals [43], antifungals [44,45,46], anti-inflammatory [47,48] and anti-tumor substances [38,43,49,50].

Additionally, chitin and its derivatives have proven to be beneficial for the wound healing process (by indirect acceleration of cultured fibroblasts, by improving the migration of fibroblasts and vascular endothelial cells, as well as the formation of granulating tissue, and by induction of stable collagen synthesis without scatter in the early wound-healing process) and have been used for the development of adhesive dressings [51].

Alginate is a preferred biodegradable natural polymer for wound dressings, due to its strong hydrophilic character that maintains a physiologically moist microenvironment, limits the wound exudate, minimizes the bacterial contamination, and can be easily rinsed with saline solution, making the changes painless and not affecting the healing granulation tissue [52,53].

Given the impressive potential biomedical applications of chitin and its derivatives, as well as the long successful use of alginate for wound dressings, we proposed within this paper the fabrication of a novel multifunctional biocompatible nanocomposite based on the combination of naturally derived chitin and sodium alginate polysaccharides and commercial antibacterial drugs (Cefuroxime and Cefepime) for enhanced wound healing process. The second and fourth generation cephalosporins antibiotics have been chosen due to their large spectrum of antimicrobial activity and previously proven good pharmacokinetics in wound secretion [54,55]. Thus, dermal tissue healing occurs due to the promising corroboration related to biocompatible and biodegradable biopolymers and antibacterial drugs.

## 2. Results

**Figure 1:**
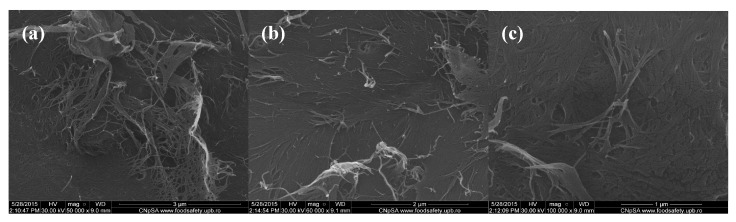
Scanning electron microscopy (SEM) images of chitin-sodium alginate composites: (**a**,**b**) 50,000×; (**c**) 100,000×.

A thermogravimetric analysis was performed in order to obtain a quantitative estimation regarding the amount of antibiotic substance that interacted with the fabricated chitin-sodium alginate nanocomposites. According to the obtained derivatogram depicted in Figure 1, one can observe two distinctive thermal processes that are specific for chitin and chitin-derivatives. The first endothermic process, which occurred until 150 °C, can be assigned to humidity water evaporation, while the second prominent thermal process, which occurred between 250 and 450 °C, is assigned to polysaccharide degradation. The second thermal process that occurred within the concerned chitin-sodium alginate composites is specifically accompanied by a significant weight loss due to biopolymer degradation, which occurs due to the depolymerization reaction (local relaxation of chitin backbone chain) and decomposition process of both acetylated and deacetylated units of chitin [56,57]. The total estimated weight loss corresponding to chitin-sodium alginate composite was 91.112% ± 0.010%, while for composite-commercial antibiotics (Cefepime or Cefuroxime) systems the determined weight loss was 92.338% ± 0.011% and 92.798% ± 0.009%, respectively. The weight losses of the antibiotics were 99.45% in the case of Cefepime and 81.52% in the case of Cefuroxime.

In order to evaluate the morphology of the fabricated composite samples based on chitin and sodium alginate and loaded with commercial antibiotics, we performed relevant scanning electron microscopy (SEM) studies. Regarding the SEM micrographs corresponding to polysaccharide composite samples with no incorporated antibiotic that are shown in Figure 1, one can observe a specific micro-fibril structure, with heterogeneous and rough aspect (thanks to the physical-guided reticulation process of sodium alginate within the chitin gel-like matrix).

**Figure 2:**
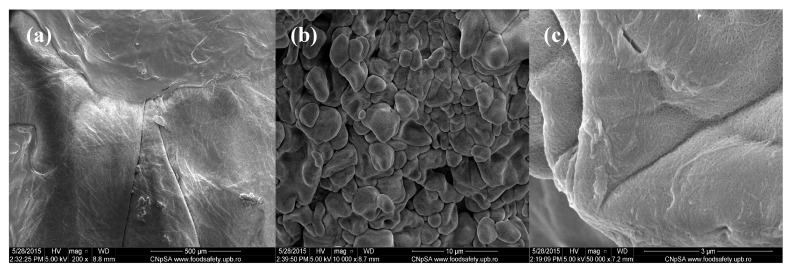
SEM images of Cefepime-loaded chitin-sodium alginate composites: (**a**) 200×; (**b**) 10,000×; (**c**) 50,000×.

The lower magnification image corresponding to the Cefepime-loaded composites that are observed in Figure 2 captures a uniform and relatively smooth microscopic aspect of the obtained samples. However, when performing higher magnification investigations (5000× and 10,000×), we can clearly observe the granular-like microstructure of the composites, with rough aspect. The identified polyhedral particles specifically possess smooth surface and micrometric dimensions (the corresponding dimensional range being between 2 μm and 5 μm) and have an increased tendency to form agglomerate structures. Moreover, if considering the 50,000× magnification of the concerned samples, one can clearly distinguish the uniform netting aspect.

**Figure 3:**
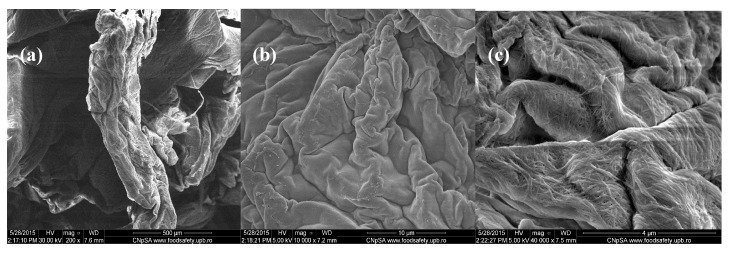
SEM images of Cefuroxime-loaded chitin-sodium alginate composites: (**a**) 200×; (**b**) 10,000×; (**c**) 40,000×.

In comparison, the images from Figure 3 corresponding to chitin-sodium alginate samples loaded with Cefuroxime antibiotic revealed a prominent irregular morphology, with a microscopic pleated aspect. Higher magnifications of the samples allowed us to observe the uniform and continuous wavy aspect of the produced drug-loaded composites (velvet-like morphology). Additionally, according to the highest magnification image (40,000× magnification), the previously observed composite structures possess rather rough surfaces, with fibril aspects.

As the scanning electron micrographs revealed, the presence of antibiotics within the chitin-sodium alginate composites significantly influences the microstructure aspects.

**Figure 4:**
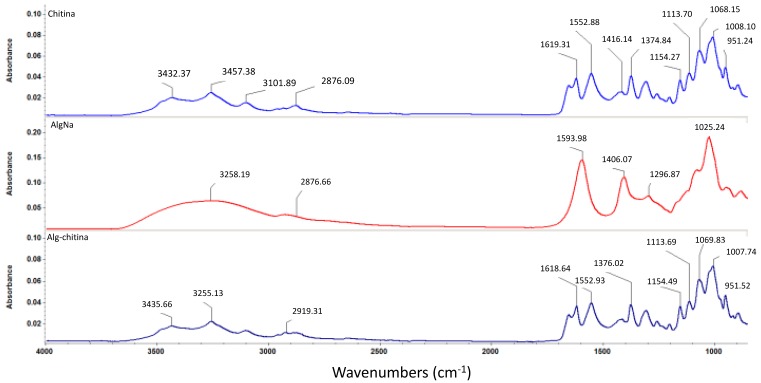
FTIR spectra corresponding to chitin, sodium alginate, and chitin-sodium alginate composite.

In order to investigate the formation of a composite structure between the two selected naturally derived polysaccharides, we performed Fourier transform infrared spectroscopic measurements, as depicted in Figure 4. One can observe that the infrared (IR) data corresponding to the composite structure maintains the same graphical aspect as the pristine chitin sample, which suggests the formation of an interconnected mixture between the selected biopolymers. However, one can observe that the identified absorbance maxima pointedly kept their allure and intensity, but the concerned wavenumbers have slightly reduced values (this behavior is due to pure physical and weak chemical interactions established between chitin and sodium alginate) [58]. As one can notice in the infrared spectrum of the biocomposite sample, specific absorbance maxima of chitin-derivatives are identified: slightly shifted bands corresponding to O–H and N–H stretching vibrations (3435 cm^−1^) and asymmetric stretching vibrations of –CH_2_– (2919 cm^−1^), N–H bending vibrations within the amide II structure at 1552 cm^−1^, and slightly shifted 1621 cm^−1^ band specific for hydrogen bond of C=O group with the hydroxymethyl group of the next chitin residue of the same chain and CH– deformation band (1376 cm^−1^) [59,60,61].

**Figure 5:**
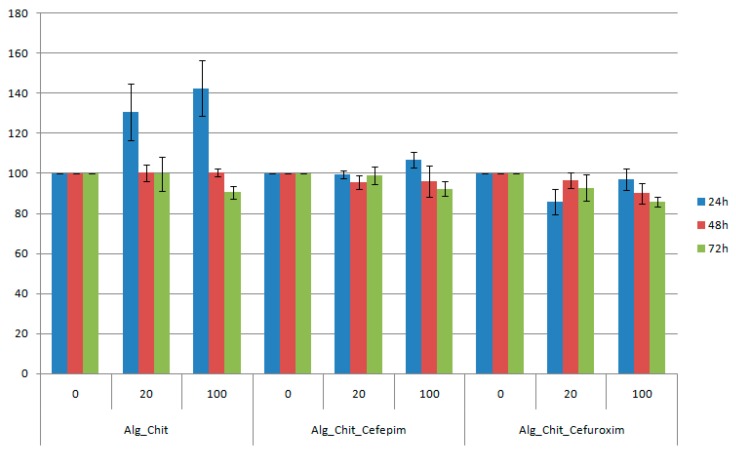
Cell viability of L929 cells incubated with undiluted and one fifth diluted chitin-based samples at 24, 48, and 72 h after the treatment. The obtained results are reported as the percentage of the untreated control. Each data point represents the mean ± SD of three experiments.

The *in vitro* bioevaluation of the chitin-based drug delivery systems was conducted on the L929 fibroblast cell line—which is commonly used in biocompatibility studies, as it is considered a highly sensitive cell line—using the tetrazolium salt-based assay (Figure 5). Regarding our study, the viability percentage was calculated by considering the untreated cells (negative control) as 100% viable.

For the 24 h treatment with chitin-sodium alginate samples extract, the L929 fibroblasts showed an accelerated metabolic processing of the tetrazolium salt, thereby suggesting an increased proliferation of the cells, with about 40% for the undiluted suspension (compared to controls). After 48 h of experimental treatment, the viability was maintained at about the same level with the control samples, while at 72 h a slight reduction of the cellular viability was noticed. Regarding the Cefepime-loaded biocomposite samples, we also observed a small proliferation effect at 24 h after the treatment, while the reported data for both 48 h and 72 h treatments are similar to pristine polysaccharide composites. However, for the Cefuroxime experimental version, the reported cellular viability was reduced by about 8% for the diluted suspension at 24 h after the treatment, reaching a maximum at 48 h, after which a decrease of 1%–2% was registered. For the undiluted suspension, the viability decreased with time. However, for all samples, dilutions, and time intervals, the viability was maintained above 80%, meaning that the samples are biocompatible (according to ISO 10993-12:2001(E) [62]).

**Figure 6:**
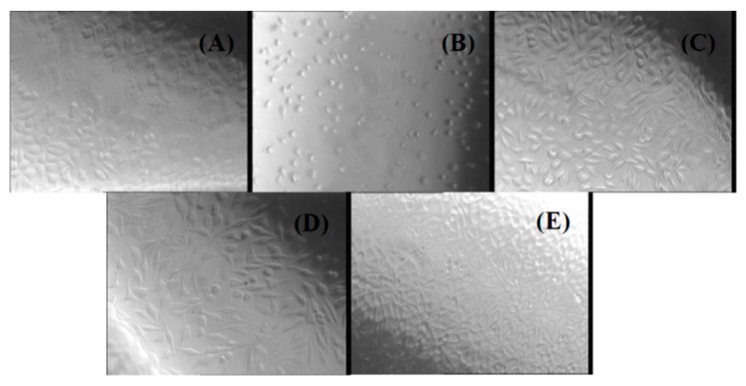
Phase contrast microscopy images for L929 cells treated with different chitin-based extracts by using the agar diffusion method: (**A**) negative control; (**B**) positive control; (**C**) NaAlg-Chitin-Cefepime; (**D**) NaAlg-Chitin-Cefuroxime; (**E**) NaAlg-Chitin.

Another method used in our study to evaluate the biocompatibility of chitin-based composites was the agar diffusion test. The cytotoxicity was evaluated by observing the degree of cellular lysis and the size of the uncolored zone around the material tested using the scoring criteria from Table 1, which contains the results of the agar diffusion test applied to a monolayer of L929 cells treated with all of the prepared experimental composites (NaAlg-Chitin, NaAlg-Chitin-Cefepime, and NaAlg-Chitin-Cefuroxime). The corresponding morphology of the fibroblasts treated with different extracts of chitin-based samples can be observed in Figure 6.

The agar diffusion test revealed that none of the three chitin-based experimental samples exerted cytotoxic effects against the treated cells, thus confirming the results obtained by the cell viability assay. The response indexes and the morphology of the cells treated with NaAlg-Chitin, NaAlg-Chitin-Cefepime, and NaAlg-Chitin-Cefuroxime are similar with those obtained for the negative control. In all cases, the L929 cellular monolayer seemed intact, without any decolorized zones due to the lysed cells. On the contrary, according to the ISO 10993 standard, the positive control proved to be moderately cytotoxic.

**Figure 7:**
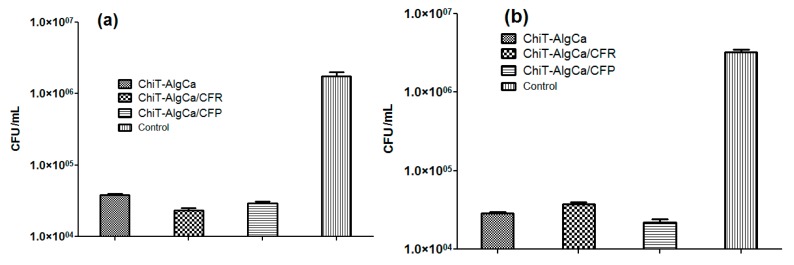
Colony forming units (CFU)/mL values for *E. coli* (**a**) and *S. aureus* (**b**) biofilms after 24 h treatment.

The *in vitro* evaluation of antimicrobial potential of antibiotic-composite samples was performed against *E. coli* and *S. aureus* bacterial strains (Figure 7). As one can observe, the pristine composites exhibited significant anti-bacterial effects against bacterial biofilm development, the reported colony forming units (CFU) in both experimental conditions being reduced with almost one and a half order of magnitude (when compared to control data). Regarding the Gram-negative strain, both antibiotic-loaded samples induced a decrease of viable biofilm-embedded cells, as revealed by the reported CFU/mL, which was lower than those obtained for the pristine material. On the other hand, for the Gram-positive cocci, the number of CFU/mL increased on the Cefuroxime-loaded composites when compared to the pristine chitin-sodium alginate samples, while the Cefepime-loaded composites showed significantly reduced values of CFU/mL. These unexpected results regarding Cefuroxime-loaded composites, taking into account that a methilicin susceptible *S. aureus* strain was used, could be explained by the fact that the amount of cefuroxime loaded in the wound dressing was inferior to the minimal required dose to eradicate the *S. aureus* biofilm. It is well known that *S. aureus* strains produce a biofilm-associated capsular polysaccharide adhesion PIA (polysaccharide intercellular adhesin) which protects the cells against antibiotics, thus requiring higher eradicating doses [63].

The antimicrobial activity of the pristine chitin-sodium alginate materials could be explained by the interaction of the cation chitin with bacterial wall lipopolysachharides of the Gram-negative cells, and, respectively, with the lipoteichoic and teichoic acids of the Gram–positive ones, leading to the destabilization of cellular membrane structure and functions and the subsequent lysis of bacterial cells.

## 3. Discussion

When it comes to designing and engineering new drug delivery platforms, some specific requirements must be considered, particularly the therapeutic efficiency maximization, with side effects minimization, as well as the biocompatibility, physical and chemical features, structural and dimensional versatility, solubility and degradability, processability and approval for medical use from specialty institutions [64,65,66].

Given the remarkable biological-related behavior and processing-related versatility of chitin and chitin-derivatives, novel biomedical systems based on this multifunctional natural polysaccharide have been recently developed and have received U.S. Food and Drug Administration commercial approval. Aquanova Ag™ and Bondiloxs™ (MedTrade Products Ltd., Crewe, UK), ChitoFlex™ PRO and ChitoGauze™ (HemCon Medical Technologies Inc. Portland, OR, USA), ExcelArrest™ XT and PosiSep™ (Hemostasis LLC Co. St Paul, MN, USA), SentrexBioSponge™ MPD (Bionova Medical Inc. Germantown, TN, USA), SoftSeal-C™ (Chitogen Inc. Plymouth, MN, USA), and Traumastat™ (Ore-Medix LLC Co. Salem, Oregon, USA) are representative examples of chitin-derived biosystems that have been developed and approved within the last ten years for wound healing applications [65]. Moreover, given the tunable features of chitin-based materials, this auspicious natural biopolymer was also investigated for novel topical healing, drug delivery, and immune enhancement applications. According to the U.S. National Institutes of Health governmental organization, there are currently few clinical trials in progress regarding the evaluation of chitin-based systems for nasal cytokine release prevention during local immune response, prevention of cardiovascular diseases by lowering both levels of pristine and oxidized low-density lipoprotein (LDL)-cholesterol, treatment of allergic rhinitis and spontaneous epistaxis, and post-partum haemorrhage management [66]. The aim of the present study was to develop a novel biocompatible nanostructured composite based on naturally derived biopolymers (chitin and sodium alginate) and commercial antibiotics (Cefuroxime and Cefepime) with dual function, namely promoting wound healing and local drug delivery. The *in vitro* cytocompatibility assays performed on L929 fibroblast revealed the lack of cytotoxicity of the obtained nanocomposites. Similar studies showed that different chitin-based composite products were found to have no toxicity on cells and to fulfil the optimal requirements of wound dressing applications (*i.e*., to be non-toxic, non-allergic, non-adherent, and sterile) [67,68]. Mori *et al*. [69] also reported that chitin and chitin-derivatives did not exhibit any acceleratory effect on the proliferation of L929 fibroblast cells. However, the cytokine secretion evaluation in rat primary cultured dermal fibroblasts exposed with chitin-based materials showed an indirect acceleration of the cells proliferation (interleukin-8 induction and no secretion of interleukin-6, 1α, 1β, nor tumor necrosis factor-α).

The antimicrobial activity assays revealed a remarkable inhibition of bacterial biofilm development, the best experimental results being obtained for the chitin-sodium alginate composites loaded with the fourth generation cephalosporin antibiotic Cefepime. Therefore, we can conclude that this antibiotic was successfully encapsulated in the polysaccharide composites.

## 4. Materials and Methods

### 4.1. Materials

In order to obtain the chitin-sodium alginate biocomposites, we used the following substances, which were purchased from Sigma Aldrich Chemie GmbH (Munich, Germany): Chitin (C_8_H_13_O_5_N)_n_, Sodium Alginate (NaAlg), Calcium chloride (CaCl_2_), Methyl alcohol (CH_4_O), Cefuroxime, and Cefepime. All required chemicals were of analytical purity and used with no further purification.

### 4.2. Synthesis

For the fabrication of novel biocompatible nanocomposites based on chitin and sodium alginate, we experimentally performed the following protocol: 45 g of CaCl_2_ were mixed with 100 mL of methanol. The as-resulted solution was filtered and further mixed with 1 g of chitin and—after 30 min of magnetic stirring—the resulted gel was placed at −20 °C for 48 h. The second polysaccharide solution was prepared by dissolving 1 g of NaAlg into 100 mL of deionized water, with the subsequent addition of the selected therapeutic agents (50 mg of Cefuroxime and 50 mg of Cefepime). The AlgNa-based aqueous solution with/without therapeutic agent content was dropwise added within the chitin-based gel, under magnetic stirring. The resulted composite materials were filtered (in order to eliminate the excess alcohol) and subjected to a multiple washing treatment with deionized water (in order to eliminate excessive calcium ions and therapeutic agents). Finally, the resulted samples were dried in ambient conditions and subjected to several investigations.

### 4.3. Characterization

#### 4.3.1. Thermogravimetric Analysis

Prior to thermogravimetric characterization, the concerned samples were sieved by using a 200 mesh screen. A reduced amount of resulted powders was placed into alumina crucibles and subjected to thermal analysis. The samples were heated in dried synthetic air (80% N_2_ and 20% O_2_) from room temperature up to 800 °C, by using a 1 °C/min heating rate. The thermogravimetric analysis was performed by using a Shimadzu DTG-TA-50H. 

#### 4.3.2. Scanning Electron Microscopy and Energy Dispersive X-ray Spectroscopy

The scanning electron microscopy and additional energy dispersive X-ray spectroscopy analyses were performed by using the secondary electron beam (30 keV) of a FEI electronic microscope equipped with Energy Dispersive X-Ray Spectroscopy (EDX).

#### 4.3.3. Fourier Transform Infrared Spectroscopy

Fourier Transform Infrared Spectroscopy (FTIR) analysis was performed by using a Nicolet iN10 MX FT-IR microscope with Mercury cadmium telluride (MCT) detector (cooled with liquid nitrogen). The spectral data acquisition was done in reflection mode at 4 cm^−1^ resolution. For each spectrum 32 scans were performed, which were further superposed and converted by using the OminoPicta (Thermo Scientific) software in order to obtain the maximum absorbance. Two hundred and fifty individual spectra were analyzed for each concerned sample.

### 4.4. Biological Evaluation

#### 4.4.1. *In Vitro* Biocompatibility Evaluation

The *in vitro* biocompatibility evaluation of the obtained chitin-based composites was assessed on the L929 fibroblast cell line, which was purchased from ATCC^®^. The cells were cultured in Earle’s minimum essential medium (MEM) (Biochrom, Merck Milipore) and supplemented with 10% fetal bovine serum (FBS) (Biochrom, Merck Milipore), 1% l-glutamine (Biochrom, Merck Milipore), and 1% penicillin and streptomycin antibiotics (Biochrom, Merck Milipore, in standard conditions (37 ± 2 °C, 5% ± 1% CO_2_, more than 90% humidity).

In order to have a quantitative estimation of cellular viability after treatment, the tetrazolium-salt (MTT—3-(4,5-dimethylthiazol-2-yl)-2,5-diphenyltetrazolium bromide) viability assay was performed. In this respect, 5000 cells/well were seeded in 96-well plates and cultured for 24 h in standard conditions. Meanwhile, extracts from the samples were prepared as following: 0.1 g/ mL of the obtained material was placed in complete culture medium in tightly closed glass containers. The extracts were obtained at 37 ± 2 °C by using an orbital shaker for 24 h. 

100 μL of the obtained extracts were further added in each of the sample wells, with 1/5 and 1/25 dilutions. In control wells (untreated cells) was added complete culture medium. Also, blank samples (wells with no cells) were prepared for the elimination of possible interferences. The viability measurements were done at 24 h, 48 h, and 72 h, respectively, after the treatment. In order to do so, the supernatant was gently removed from the wells and 10 μL of MTT solution was added over 90 μL of culture medium (complete MEM, supplemented with 5% FBS) in each well. The plates were incubated for 3 h in standard conditions and the corresponding absorbance was read at 570 nm using a Mitrasmicroplate reader.

The agar diffusion assay for biocompatibility assessment was performed according to the ISO 10993 standard. 200,000 cells/well were seeded in gridded Petri dishes (three for each sample, three negative controls, and three positive controls) and cultured in standard conditions for 24 h. Subsequently, a 1% noble Agar solution was mixed with MEM 2x medium in equal amounts and used to replace the culture medium inside the previously prepared Petri dishes. The samples were incubated to allow the gelling of the Agar medium and a Milipore disk soaked in extract/culture medium/0.45% phenol solution was placed on each corresponding Petri dish (samples/negative control/positive control). After another 24 h in standard conditions, the cells were colored with Neutral Red solution, incubated for 1 h in standard conditions, and counted according to the standard specifications. The degree of cellular lysis and the morphology of as-treated fibroblast-like cells was evaluated by using a phase contrast OLYMPUS microscope (40× magnification), as described in our previous paper [70].

All the experiments were performed in triplicate and repeated three times.

#### 4.4.2. *In Vitro* Antimicrobial Activity Assay

In order to evaluate the anti-bacterial and anti-adherent potential of the obtained chitin-based biocompatible nanostructured composites, *in vitro* assays against two clinically relevant bacterial strains were performed—namely *Escherichia coli* and *Staphylococcus aureus*—by using the viable cell count assay.

Pristine and antibiotic-loaded composites sections were prepared and individually placed within 6-well plates, which were further inoculated with 2 mL suspensions of 0.5 McFarland standard density (1.5 × 10^8^ CFU/mL) of *E. coli* and *S. aureus*.

After 24 h incubation at 37 °C, the biomaterial sections were gently washed with sterile phosphate buffered saline (PBS) solution and transferred into 1.5 mL centrifuge tubes with 750 μL of PBS. In order to disperse the biofilm-forming bacterial cells, the concerned samples were centrifuged for 30 s and sonicated for 10 s. Serial ten-fold dilutions were prepared from each solution and 100 mL corresponding to the final dilution were transferred to Luria-Broth (LB) agar-containing Petri plates for viable cells count assay [71,72]. All the experiments were performed in triplicate and repeated three times.

#### 4.4.3. Statistics

The reported results are presented as means ± standard error of the mean. Data were statistically analyzed by using a two-tailed Student’s test. *p* values ≤ 0.05 were accepted as statistically significant.

## 5. Conclusions

The chitin-based materials synthetized in the present study with a proved biocompatibility and antimicrobial properties seem to be promising for use in the management of wound healing.

## Figures and Tables

**Table 1:** Cytotoxicity evaluation using agar diffusion test-zone description.

Index		Zone Description/Score—Cytotoxicity Degree	Negative Control	Positive Control	NaAlg-Chitin-Cefepim	NaAlg-Chimin-Cefuroxim	NaAlg-Chitin
Zone	0	No detectable zone around or under specimen	0		0	0	0
	1	Zone limited to the area under specimen					
	2	Zone extends less than 0.5 cm beyond specimen		2			
	3	Zone extends between 0.5 cm and 1.0 cm beyond specimen					
	4	Zone extends greater than 1.0 cm beyond specimen, but does not involve the entire culture dish					
	5	Zone involves entire culture dish					
Lysis	0	No observable cytotoxicity					
	1	Less than 20% of zone affected	1		1	1	1
	2	20% to 39% of zone affected					
	3	40% to 59% of zone affected		3			
	4	60% to 80% of zone affected					
	5	Greater than 80% of zone affected					
Index	0	0/0–0.5/0.5–0/1Non-cytotoxic	0/1		0/1	0/1	0/1
	1	1/1–1.5/1.5—Mild					
	2	2/2–3/—Moderate		2/3			
	3	>3/4—Severe					
Results			Non-cytotoxic	Moderate	Non-cytotoxic	Non-cytotoxic	Non-cytotoxic

## References

[1] Wei X., Chen X., Ying M., Lu W. (2014). Brain tumor-targeted drug delivery strategies. Acta Pharm. Sin. B.

[2] Hastings C.L., Roche E.T., Ruiz-Hernandez E., Schenke-Layland K., Walsh C.J., Duffy G.P. (2015). Drug and cell delivery for cardiac regeneration. Adv. Drug Deliv. Rev..

[3] Bruix J., Han K.H., Gores G., Llovet J.M., Mazzaferro V. (2015). Liver cancer: Approaching a personalized care. J. Hepatol..

[4] Zhou P., Sun X., Zhang Z. (2014). Kidney-targeted drug delivery systems. Acta Pharm. Sin. B.

[5] Hua S., Marks E., Schneider J.J., Kelly S. (2015). Advances in oral nano-delivery systems for colon targeted drug delivery in inflammatory bowel disease: Selective targeting to diseased *versus* healthy tissue. Nanomed. Nanotechnol. Biol. Med..

[6] Farinha I., Duarte P., Pimentel A., Plotnikova E., Chagas B., Mafra L., Grandfils C., Freitas F., Fortunato E., Reis M.A.M. (2015). Chitin-glucan complex production by Komagataella pastoris: Downstream optimization and product characterization. Carbohydr. Polym..

[7] Kaya M., Baublys V., Šatkauskienė I., Akyuza B., Bulut E., Tubelytė V. (2015). First chitin extraction from Plumatellarepens (Bryozoa) with comparison to chitins of insect and fungal origin. Int. J. Biol. Macromol..

[8] Lu H., Lu L., Zeng L., Fu D., Xiang H., Yu T., Zheng H. (2014). Effect of chitin on the antagonistic activity of Rhodosporidiumpaludigenum against Penicilliumexpansum in apple fruit. Postharvest Biol. Technol..

[9] Roca C., Chagas B., Farinha I., Freitas F., Mafra L., Aguiar F., Oliveira R., Reis M.A.M. (2012). Production of yeast chitin–glucan complex from biodiesel industry byproduct. Process. Biochem..

[10] Chandran R., Williams L., Hung A., Nowlin K., LaJeunesse D. (2016). SEM characterization of anatomical variation in chitin organization in insect and arthropod cuticles. Micron.

[11] Shi J.F., Fu J., Mu L.L., Guo W.C., Li G.Q. (2016). Two Leptinotarsa uridine diphosphate N-acetylglucosaminepyrophosphorylases are specialized for chitin synthesis in larval epidermal cuticle and midgut peritrophic matrix. Insect Biochem. Mol. Biol..

[12] Kaya M., Erdogan S., Mol A., Baran T. (2015). Comparison of chitin structures isolated from seven Orthoptera species. Int. J. Biol. Macromol..

[13] Hafsa J., Smach M.A., Charfeddine B., Limem K., Majdoub H., Rouatbi S. (2016). Antioxidant and antimicrobial proprieties of chitin and chitosan extracted from ParapenaeusLongirostris shrimp shell waste. Ann. Pharm. Fr..

[14] Osada M., Miura C., Nakagawa Y.S., Kaihara M., Nikaido M., Totani K. (2015). Effect of sub- and supercritical water treatments on the physicochemical properties of crab shell chitin and its enzymatic degradation. Carbohydr. Polym..

[15] Hajji S., Ghorbel-Bellaaj O., Younes I., Jellouli K., Nasri M. (2015). Chitin extraction from crab shells by Bacillus bacteria. Biological activities of fermented crab supernatants. Int. J. Biol. Macromol..

[16] Salaberria A.M., Herrera Diaz R., Labidi J., Fernandes S.C.M. (2015). Preparing valuable renewable nanocomposite films based exclusively on oceanic biomass—Chitin nanofillers and chitosan. React. Funct. Polym..

[17] Shushizadeh M.R., Pour E.M., Zare E., Lashkari Z. (2015). Persian gulf β-chitin extraction from sepia pharaonis sp. cuttlebone and preparation of its derivatives. Bioact. Carbohydr. Diet. Fibre.

[18] Bogdanova O.I., Polyakov D.K., Streltsov D.R., Bakirov A.V., Blackwell J., Chvalun S.N. (2016). Structure of β-chitin from Berryteuthis magister and its transformation during whisker preparation and polymerization filling. Carbohydr. Polym..

[19] Vázquez J.A., Caprioni R., Nogueira M., Menduiña A., Ramos P., Pérez-Martínc R.I. (2015). Valorisation of effluents obtained from chemical and enzymatic chitin production of Illexargentinus pen by-products as nutrient supplements for various bacterial fermentations. Biochem. Eng. J..

[20] Jung J., Zhao Y. (2014). Alkali- or acid-induced changes in structure, moisture absorption ability and deacetylating reaction of β-chitin extracted from jumbo squid (Dosidicusgigas) pens. Food Chem..

[21] Wan A.C.A., Tai B.C.U. (2013). CHITIN—A promising biomaterial for tissue engineering and stem cell technologies. Biotechnol. Adv..

[22] Anitha A., Sowmya S., Kumar P.T.S., Deepthi S., Chennazhi K.P., Ehrlich H., Tsurkan M., Jayakumar R. (2014). Chitin and chitosan in selected biomedical applications. Prog. Polym. Sci..

[23] Rolandi M., Rolandi R. (2014). Self-assembled chitin nanofibers and applications. Adv. Colloid Interface Sci..

[24] Naba A., Clauser K.R., Ding H., Whittaker C.A., Carr S.A., Hynes R.O. (2015). The extracellular matrix: Tools and insights for the “omics” era. Matrix Biol..

[25] Watson W.H., Ritzenthaler J.D., Roman J. (2016). Lung extracellular matrix and redox regulation. Redox Biol..

[26] Theocharis A.D., Skandalis S.S., Gialeli C., Karamanos N.K. (2016). Extracellular matrix structure. Adv. Drug Deliv. Rev..

[27] Vishnu Priya M., Sabitha M., Jayakumar R. (2016). Colloidal chitin nanogels: A plethora of applications under one shell. Carbohydr. Polym..

[28] Hinderer S., Layland S.L., Schenke-Layland K. (2016). ECM and ECM-like materials—Biomaterials for applications in regenerative medicine and cancer therapy. Adv. Drug Deliv. Rev..

[29] Wang C., Esker A.R. (2014). Nanocrystalline chitin thin films. Carbohydr. Polym..

[30] Dubey L.K., Moeller J.B., Schlosser A., Sorensen G.L., Holmskov U. (2015). Chitin enhances serum IgE in Aspergillus fumigatus induced allergy in mice. Immunobiology.

[31] Jayakumar R., Nair A., Rejinold N.J., Maya S., Nair S.V. (2012). Doxorubicin-loaded pH-responsive chitin nanogels for drug delivery to cancer cells. Carbohydr. Polym..

[32] Arunraj T.R., Rejinold N.S., Kumar N.A., Jayakumar R. (2013). Doxorubicin-chitin-poly(caprolactone) composite nanogel for drug delivery. Int. J. Biol. Macromol..

[33] Keinath N.F., Waadt R., Brugman R., Schroeder J.I., Grossmann G., Schumacher K., Krebs M. (2015). Live Cell Imaging with R-GECO1 Sheds Light on flg22- and Chitin-Induced Transient [Ca^2+^]cyt Patterns in Arabidopsis. Mol. Plant.

[34] Liu H., Yang Q., Zhang L., Zhuo R., Jiang X. (2016). Synthesis of carboxymethyl chitin in aqueous solution and its thermo- and pH-sensitive behaviors. Carbohydr. Polym..

[35] Izumi R., Komada S., Ochi K., Karasawa L., Osaki T., Murahata Y., Tsuka T., Imagawa T., Itoh N., Okamoto Y. (2014). Favorable effects of superficially deacetylated chitin nanofibrils on the wound healing process. Carbohydr. Polym..

[36] Kumar R.A., Sivashanmugam A., Deepthi S., Bumgardner J.D., Nair S.V., Jayakumar R. (2016). Nano-fibrin stabilized CaSO4 crystals incorporated injectable chitin composite hydrogel for enhanced angiogenesis & osteogenesis. Carbohydr. Polym..

[37] Smith K.T., Anitha A., Furuike T., Tamura H., Nair S.V., Jayakumar R. (2013). In vitro evaluation of paclitaxel loaded amorphous chitin nanoparticles for colon cancer drug delivery. Colloids Surf. B Biointerfaces.

[38] Shang Y., Ding F., Xiao L., Deng H., Du Y., Shi X. (2014). Chitin-based fast responsive pH sensitive microspheres for controlled drug release. Carbohydr. Polym..

[39] Geetha P., Sivaram A.J., Jayakumar R., Mohan C.P. (2016). Integration of in silico modeling, prediction by binding energy and experimental approach to study the amorphous chitin nanocarriers for cancer drug delivery. Carbohydr. Polym..

[40] Smitha K.T., Nisha N., Maya S., Biswas R., Jayakumar R. (2015). Delivery of rifampicin-chitin nanoparticles into the intracellular compartment of polymorphonuclear leukocytes. Macromolecules.

[41] Dutta A.K., Egusa M., Kaminaka H., Izawa H., Morimotoa M., Saimoto H., Ifuku S. (2015). Facile preparation of surface N-halamine chitin nanofiber to endow antibacterial and antifungal activities. Carbohydr. Polym..

[42] Chien R.C., Yen M.T., Mau J.L. (2016). Antimicrobial and antitumor activities of chitosan from shiitake stipes, compared to commercial chitosan from crab shells. Carbohydr. Polym..

[43] Yao Q., Wu C.F., Luo P., Xiang X.C., Liu J.J., Mou L., Bao K.J. (2010). A new chitin-binding lectin from rhizome of Setcreaseapurpurea with antifungal, antiviral and apoptosis-inducing activities. Process. Biochem..

[44] Salaberria A.M., Fernandes S.C.M., Herrera Diaz R., Labidi J. (2015). Processing of α-chitin nanofibers by dynamic high pressure homogenization: Characterization and antifungal activity against A. niger. Carbohydr. Polym..

[45] Ifuku S., Tsukiyama Y., Yukawa T., Egusa M., Kaminaka H., Izawa H., Morimoto M., Saimoto H. (2015). Facile preparation of silver nanoparticles immobilized on chitin nanofiber surfaces to endow antifungal activities. Carbohydr. Polym..

[46] Robles E., Salaberria A.M., Herrera R., Fernandes S.C.M., Labidi J. (2016). Self-bonded composite films based on cellulose nanofibers and chitin nanocrystals as antifungal materials. Carbohydr. Polym..

[47] Kumar P.T.S., Ramya C., Jayakumar R., Nair S.K.V., Lakshmanan V.K. (2013). Drug delivery and tissue engineering applications of biocompatible pectin–chitin/nano CaCO_3_ composite scaffolds. Colloids Surf. B Biointerfaces.

[48] Ghotloo S., Hoseini M.H.M., Alimohammadian M.H., Khaze V., Memarnejadian A., Rostami A. (2015). Immunomodulatory effects of chitin microparticles on Leishmania major-infected BALB/c mice. Parasitol. Int..

[49] Jain T., Kumar S., Dutta P.K. (2016). Dibutyrylchitin nanoparticles as novel drug carrier. Int. J. Biol. Macromol..

[50] Prabha G., Raj V. (2016). Preparation and characterization of polymer nanocomposites coated magnetic nanoparticles for drug delivery applications. J. Magn. Magn. Mater..

[51] Azuma K., Izumi R., Osaki T., Ifuku S., Morimoto M., Saimoto H., Minami S., Okamoto Y. (2015). Chitin, Chitosan, and Its Derivatives for Wound Healing: Old and New Materials. J. Funct. Biomater..

[52] Gilchrist T., Martin A.M. (1983). Wound treatment with sorbsan—An alginate fibre dressing. Biomaterials.

[53] Motta G.J. (1989). Calcium alginate topical wound dressings: A new dimension in the cost-effective treatment for exudating dermal wounds and pressure sores. Ostomy Wound Manag..

[54] Bischoff M., Beck A., Frei P., Bischoff G. (2010). Pharmacokinetics of cefuroxime in traumatic wound secretion and antibacterial activity under vacuum therapy. J. Chemother. (Florence, Italy).

[55] Gentry L.O., Rodriguez-Gomez G. (1991). Randomized comparison of cefepime and ceftazidime for treatment of skin, surgical wound, and complicated urinary tract infections in hospitalized subjects. Antimicrob. Agents Chemother..

[56] Georgieva V., Zvezdova D., Vlaev L. (2013). Non–isothermal kinetics of degradation of chitin and chitosan. J. Therm. Anal. Calorim..

[57] Praveen P., Rao V. (2014). Synthesis and Thermal Studies of Chitin/AgCl nanocomposite. Procedia Mater. Sci..

[58] Lee K.Y., Mooney D.J. (2012). Alginate: Properties and biomedical applications. Prog. Polym. Sci..

[59] Rinaudo M. (2006). Chitin and chitosan: Properties and applications. Prog. Polym. Sci..

[60] Beil S., Schamberger A., Naumann W., Machill S., van Pée K.H. (2012). Determination of the degree of N-acetylation (DA) of chitin and chitosan in the presence of water by first derivative ATR FTIR spectroscopy. Carbohydr. Polym..

[61] Sayari N., Sila A., Abdelmalek B.A., Abdallah R.B., Ellouz-Chaabouni S., Bougatef A., Balti R. (2016). Chitin and chitosan from the Norway lobster by-products: Antimicrobial and anti-proliferative activities. Int. J. Biol. Macromol..

[62] International Organization for Standardization (ISO) (2016). 10993—Biological Evaluation of Medical Devices.

[63] Götz F. (2002). Staphylococcus and biofilms. Mol. Microbiol..

[64] Kang L., Gao Z., Huang W., Jin M., Wang Q. (2015). Nanocarrier-mediated co-delivery of chemotherapeutic drugs and gene agents for cancer treatment. Acta Pharm. Sin. B.

[65] US Food and Drug Administration. http://www.fda.gov/.

[66] Clinical Trials, a service of the US National Institutes of Health. https://clinicaltrials.gov/.

[67] Singh R., Chacharkar M.P., Mathur A.K. (2008). Chitin membrane for wound dressing application—Preparation, characterisation and toxicological evaluation. Int. Wound J..

[68] Kumar P.T.S., Abhilash S., Manzoor K., Nair S.V., Tamura H., Jayakumar R. (2010). Preparation and characterization of novel b-chitin/nanosilver composite scaffolds for wound dressing applications. Carbohydr. Polym..

[69] Mori T., Okumura M., Matsuura M., Ueno K., Tokura S., Okamoto Y., Minami S., Fujinaga T. (1997). Effects of chitin and its derivatives on the proliferation and cytokine production of fibroblasts *in vitro*. Biomaterials.

[70] Grigore F., Lungu M., Savu D., Radu M., Velciu G. (2012). Preparation, characterization and biological evaluation of tricalcium phosphate granules. Romanian J. Mater..

[71] Lehtinen J. (2007). Improvements in the Assessment of Bacterial Viability and Killing. Ph.D. Thesis.

[72] Mihaiescu D.E., Grumezescu A.M., Andronescu E., Voicu G., Ficai A., Vasile O.R., Bleotu C., Saviuc C. (2013). Prosthetic Devices with Functionalized antibiofilm surface based NanoAg@C18. Curr. Org. Chem..

